# “Left Neglected,” but Only in Far Space: Spatial Biases in Healthy Participants Revealed in a Visually Guided Grasping Task

**DOI:** 10.3389/fneur.2014.00004

**Published:** 2014-01-22

**Authors:** Natalie de Bruin, Devon C. Bryant, Claudia L. R. Gonzalez

**Affiliations:** ^1^Brain in Action Laboratory, Department of Kinesiology and Physical Education, University of Lethbridge, Lethbridge, AB, Canada

**Keywords:** pseudoneglect, visuospatial neglect, attention, human, peripersonal space, reach-to-grasp, handedness

## Abstract

Hemispatial neglect is a common outcome of stroke that is characterized by the inability to orient toward, and attend to stimuli in contralesional space. It is established that hemispatial neglect has a perceptual component, however, the presence and severity of motor impairments is controversial. Establishing the nature of space use and spatial biases during visually guided actions amongst healthy individuals is critical to understanding the presence of visuomotor deficits in patients with neglect. Accordingly, three experiments were conducted to investigate the effect of object spatial location on patterns of grasping. *Experiment 1* required right-handed participants to reach and grasp for blocks in order to construct 3D models. The blocks were scattered on a tabletop divided into equal size quadrants: left near, left far, right near, and right far. Identical sets of building blocks were available in each quadrant. Space use was dynamic, with participants initially grasping blocks from right near space and tending to “neglect” left far space until the final stages of the task. *Experiment 2* repeated the protocol with left-handed participants. Remarkably, left-handed participants displayed a similar pattern of space use to right-handed participants. In *Experiment 3* eye movements were examined to investigate whether “neglect” for grasping in left far reachable space had its origins in attentional biases. It was found that patterns of eye movements mirrored patterns of reach-to-grasp movements. We conclude that there are spatial biases during visually guided grasping, specifically, a tendency to neglect left far reachable space, and that this “neglect” is attentional in origin. The results raise the possibility that visuomotor impairments reported among patients with right hemisphere lesions when working in contralesional space may result in part from this inherent tendency to “neglect” left far space irrespective of the presence of unilateral visuospatial neglect.

## Introduction

Successful action and interaction with the environment are dependent on correctly perceiving the space around us as well as the objects within that space. In our daily lives we interact with and manipulate objects which are nearby; for example, picking up a glass of water at the dinner table. We also interact with objects which are further away by moving to the target, changing posture, or using a tool to bring the object within working space. Accordingly, space is typically behaviorally differentiated into peripersonal and extrapersonal space. Peripersonal space is commonly defined as the space immediately surrounding the body in which hand and arm actions on objects can be performed most effectively (1). In contrast, extrapersonal space refers to the space beyond peripersonal space (2). Interactions with an object in extrapersonal space would require a person to physically move toward the object, or the object would need to be moved toward the person. Impairments of spatial perception can have a devastating effect on our functional independence and quality of life.

A relatively common acquired disorder of spatial perception is hemispatial neglect which is characterized by deficits in the ability to respond to, orient toward, and attend to stimuli presented in contralesional (typically the left side of) space despite intact basic motor and sensory functions (3). A number of clinical tests are commonly used to assess the presence, severity, and progression of neglect, including line bisection tasks, target cancelation, target detection, and drawing and copying tasks. These tasks are normally completed in peripersonal space with neglect patients (following right hemisphere stroke) typically displaying a rightward bias of veridical midpoint in the line bisection task, decreasing target detection from right to left in the target cancelation and detection tasks, and a drawing which is incomplete on the left hand side in the copying task (4–10). Double dissociations of neglect symptoms have, however, been reported between contralesional peripersonal and extrapersonal space for the line bisection and task cancelation tasks. In some patients the rightward bias is present only in peripersonal space and is attenuated or extinguished in extrapersonal space, conversely other patients show more severe neglect in extrapersonal space than in peripersonal space (11–17). Dissociation between peripersonal and extrapersonal space has also been observed amongst neurologically intact adults when using the line bisection task. In contrast to neglect patients, however, healthy adults typically display a systematic leftward displacement of the line midpoint from true center when completing the task in peripersonal space, a phenomenon which is commonly referred to as *pseudoneglect* (18, 19). While some studies have failed to find an effect of distance (i.e., peripersonal vs. extrapersonal space) in *pseudoneglect* (14, 20) other studies have reported that when working in extrapersonal space, healthy participants display similar rightward shifts of bisection as patients with neglect (21–25). Collectively, these observations suggest a functional and neural dissociation between the coding of near and far space in humans.

Despite the apparent simplicity of the target cancelation and line bisection tasks, they are both complex activities in which perceptual and motor factors are generally implicated. While the severe perceptual deficits experienced by people living with hemispatial neglect have been extensively studied and well documented [see Ref. (26–28) for review], the presence, direction, and severity of visuomotor impairments, particularly in contralesional space, is less clear. Numerous studies have reported visuomotor difficulties that parallel the perceptual impairments of neglect patients (29–35), while other studies have shown normal (or near normal) visuomotor performance in reaching and grasping tasks on both sides of space amongst neglect patients (36–39). Methodological considerations including the common omission of a patient group with right hemisphere lesions but without neglect continue to contribute to the ongoing controversy surrounding visuomotor performance amongst individuals with neglect (40); however, thus far, an inimitable explanation for the divergence in the literature has yet to be determined. It is necessary to first establish the nature of space use and potential spatial biases during goal-directed visually guided actions amongst healthy individuals before we can fully understand the presence, severity, and ultimately the rehabilitation and treatment of visuomotor deficits in patients with neglect.

Accordingly, the purpose of the present series of studies was to characterize space use during an ecologically valid visually guided grasping task in healthy adults. The task involved reaching for and grasping building blocks scattered on a tabletop in order to replicate a series of 3D models (41, 42). The tabletop was notionally divided into equal size quadrants differentiated into left and right hemispace and near and far reachable space (left near, left far, right near, and right far). The blocks necessary to build each model were available in each of the quadrants (i.e., equivalent characteristics for each quadrant). The grasping task was conducted amongst right-handed (*Experiment 1*) and left-handed (*Experiment 2*) participants, allowing us to determine whether handedness plays a role in the patterns of space use. The experiment was subsequently repeated amongst right-handed participants fitted with eye tracking glasses (*Experiment 3*) allowing us to investigate whether spatial biases observed during the grasping task were attentional in origin.

## Experiment 1: Right-Handers

### Materials and methods

#### Participants

Sixteen self-reported right-handed participants were recruited from the University of Lethbridge student population to take part in *Experiment 1* (six males; 18–35 years). Participant gender was not balanced, as gender differences have not been reported in earlier studies involving a similar task (43). The study was performed with approval by the University of Lethbridge Human Subject Research Committee. Written informed consent was provided prior to the initiation of the study. Participants were naïve to the purposes of the study.

#### Apparatus and stimuli

##### Handedness questionnaire

Participants completed a modified version of the Edinburgh (44) and Waterloo (45) handedness questionnaires upon completion of the building block task. This modified handedness questionnaire included questions on hand preference for 22 different activities, with participants identifying which hand they prefer to use for each activity [see Ref. (42) for complete description].

##### Block building task

**Figure 1:**
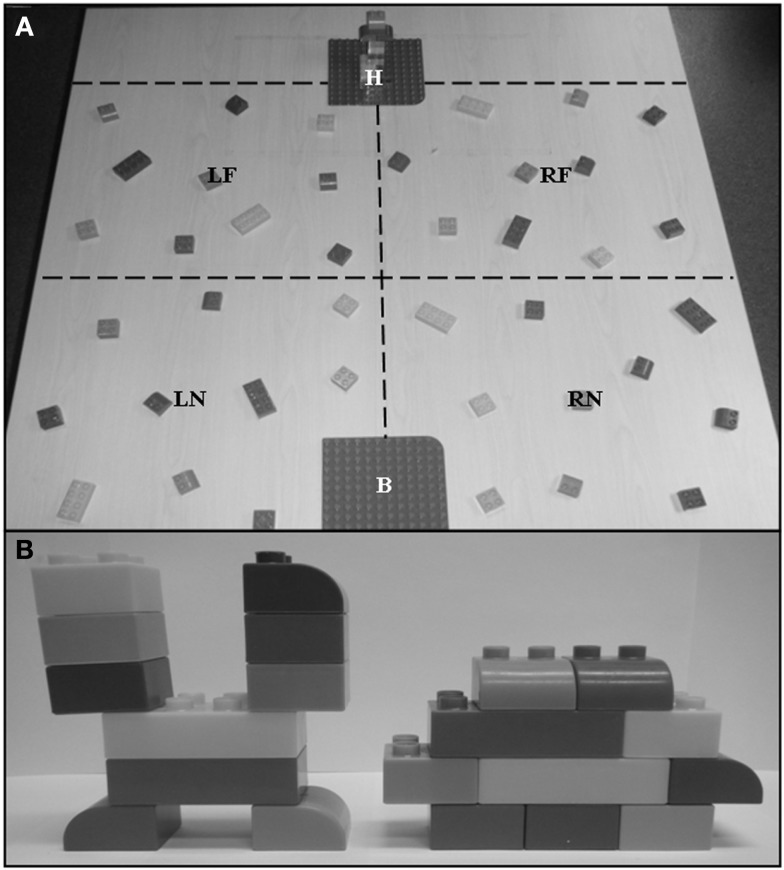
**Experimental set-up**. **(A)** Example of workspace prior to first trial. Red dashed lines notionally divide the workspace into quadrants; left near (LN), left far (LF), right near (RN), and right far (RF) reachable space. Model to be replicated is located on far base plate “H” positioned between LF and RF quadrants. Model to be constructed on near base plate “B” positioned between LN and RN quadrants. **(B)** Examples of 10-piece models. Workspace set-up and models are illustrated with MEGA BLOKS^®^.

Participants were instructed to construct a total of eight models; four using MEGA BLOKS^®^ and four using LEGO^®^ blocks (ranging in size from <0.7 L × 0.7 W × 1.0 cm H to 6.3 L × 3.1 W × 2.0 cm H). Each model was constructed from 10 blocks, which varied in color, size, and/or shape (for a total of 40 blocks per set of 4 models). The blocks for one set of four models were distributed within the workspace (70 L × 122 W × 74 cm H) which was notionally divided into equal sized quadrants demarcated by left (LEFT) and right (RIGHT) hemispace, as well as near and far reachable space. Near reachable space (NEAR) was defined as the space within reach of either hand without trunk flexion (approximately 0–35 cm), whereas far reachable space (FAR) was the workspace beyond the limits of actable space without trunk flexion (approximately 35–70 cm). These limits were adjusted for each participant to account for body/arm length. Each participant sat on the chair in front of the table and was asked to fully extend his/her arms (without trunk flexion). The point on the table at which the tip of the fingers reached was considered the limit of NEAR and the beginning of the FAR reachable space. The outer boundary of FAR space was such that it represented the furthest reachable space with trunk flexion and full arm extension (approximately 70 cm). There were no visible demarcations in the workspace that would cue participants that space use was the variable of interest. One set of the same 10 blocks necessary to complete a single model was randomly distributed into each quadrant of the workspace (Figure 1A); participants were unaware of this manipulation.

#### Procedures

Participants were seated centrally in front of the table (122 L × 122 W × 74 cm H) at a normalized distance such that when the arms were fully extended the fingertips would reach the notional division between NEAR and FAR reachable space. Consequently, a change in posture (i.e., trunk flexion) was necessary in order to grasp the blocks in FAR (reachable) space. The first model to be replicated was placed on a base plate located centrally at the far junction between left and right space (Figure 1A). Participants were requested to replicate the displayed model as quickly and accurately as possible on a second base plate (19 L × 19 W cm) located centrally immediately in front of the participant (at the intersection of right and left space; Figure 1A) from the blocks distributed on the tabletop. No further instructions were provided. As such, participants were free to use either or both hands to grasp blocks, construct, and stabilize the model. Prior research (42) using the task has highlighted the bimanual nature of the task, however, no specific instruction as to hand use were provided. Following replication of the sample model, both models were removed from the table and a new model to be replicated was provided, this process was repeated until a set of four models had been completed. Building blocks were not replaced between trials, but were replaced between each set of four models. The same eight models were used for each participant (see Figure 1B for examples); model order was randomized between participants.

The total time taken to complete each trial (i.e., search and construction) was recorded using a stopwatch. In addition, model construction was recorded for subsequent analysis using a digital video camera (JVC HD Everio^®^) placed directly in front of the participants (approximately 160 cm away from participant) with a clear view of the workspace, building blocks, and participants’ hands.

#### Data processing and analysis

All video recordings were analyzed offline. Each grasp was scored manually as a left- or right-handed grasp to ipsilateral or contralateral space. The total number of grasps was also calculated to allow the determination of the percentage of right hand use [(number of grasps with right hand/total number of grasps) × 100]. In addition, the videos of the construction of the first and fourth model in each model set were manually scored to provide the number of building blocks removed from each quadrant for each model. Model 1 provides information on space use when there is equal opportunity to grasp blocks from any quadrant of space while Model 4 offers data on the space attended to (i.e., grasped from) last. To provide a more detailed indication of space use, participant grasps were numbered in the order of occurrence (1–40) and that number was allocated to the appropriate quadrant. Each set of four models yielded a sum grasp total of 820 for the 40 blocks. The minimum possible grasp total for a quadrant was 55, which would indicate that all 10 blocks for the first model (grasps 1–10; 1 + 2 + 3 + 4 + 5 + 6 + 7 + 8 + 9 + 10 = 55) were selected from the same quadrant. The highest possible grasp total for a quadrant was 355, indicating that all 10 blocks for the fourth model (grasps 31–40; 31 + 32 + 33 + 34 + 35 + 36 + 37 + 38 + 39 = 355) were selected from the same quadrant. Within a quadrant, a grasp total between 55 and 355 would indicate that the blocks in that quadrant were selected over the course of more than one model. Lower numbers indicate that blocks from that quadrant were grasped earlier in the construction of the model set; higher numbers indicate that blocks were generally grasped later in model construction. Data were averaged across model sets.

Data were analyzed using SPSS Statistics 18.0 for Windows (SPSS Inc., Chicago, IL, USA). Statistical significance was set at α = 0.05 unless otherwise stated. Effect size (ES) was reported as η^2^ values. Handedness questionnaire and hand use data were summarized descriptively. The percentage of contralateral grasps made with each hand over the course of model set construction was assessed using paired samples *t*-tests. Trial times were entered into a one-way repeated measures analyses of variance (RM ANOVA), with Models 1–4 as a within-subject factor. When statistical significance was reported Bonferroni corrected pairwise comparisons were performed between all model pairs (*p* ≤ 0.008). Space use data for the first and fourth models (based on blocks used) were entered into separate two-factor (hemispace × distance) RM ANOVAs, with hemispace (LEFT, RIGHT), and distance (NEAR, FAR) as within-subject factors. Bonferroni corrected pairwise comparisons were performed between the near space quadrants (LN and RN) and the far space quadrants (LF and RF) pairs (*p* ≤ 0.025) when statistical significance was established. Similarly, overall space use as determined by grasp total scores was analyzed using a two-factor (hemispace × distance) RM ANOVA. Subsequently, Bonferroni corrected planned pairwise comparisons were performed between left far (LF) space and left near (LN), right near (RN), and right far (RF) space (*p* ≤ 0.017).

### Results

#### Handedness questionnaire

All participants self-reported as right-handed; this was confirmed by the handedness questionnaire score. The average handedness questionnaire score was +34.8 ± 4.9 (scores ranging from +22 to +41) where +44 would indicate exclusive right hand use for the identified activities (−44 would indicate exclusive left hand use).

#### Trial times

Trial times were significantly affected by the model being constructed [*F*(3,45) = 4.922, *p* = 0.005, ES = 0.247], with participants completing the final model (Model 4) significantly faster than the first [*t*(15) = 4.724, *p* < 0.001] and third [*t*(15) = 3.653, *p* = 0.002] models.

#### Hand use for grasping

Overall, participants used their dominant right hand for 69.5 ± 13.9% of all grasps. Analysis of contralateral grasps showed that participants used their left hand significantly less than their right hand [*t*(14) = 5.488, *p* < 0.001] when grasping in contralateral space (right hand = 21.9 ± 12.8%; left hand = 2.4 ± 1.73%).

#### Space use

##### First model

**Figure 2:**
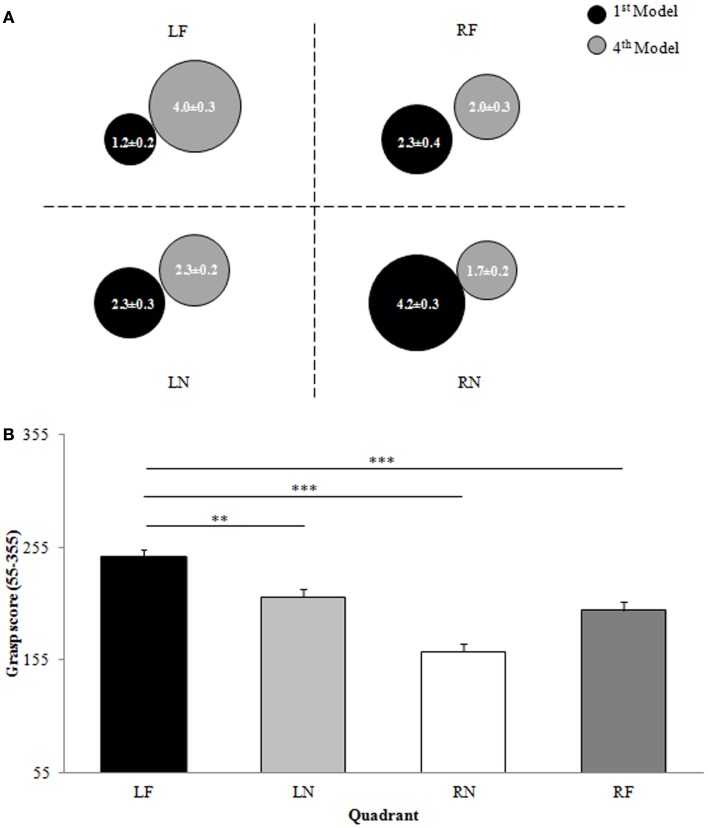
**Experiment 1: right-handers**. **(A)** Representation of the proportion of blocks (0–10) used from each quadrant for the construction of the first and fourth models in the model set. Data are means and standard errors. **(B)** Overall grasp score for each quadrant of space. Data are means and standard errors. LF, left far; LN, left near; RN, right near; RF, right far. *p < 0.05, **p < 0.01, ***p < 0.001 with respect to LF.

Participants grasped 6.5 ± 1.5 blocks from right and 3.5 ± 1.5 blocks from left hemispace to construct the first model, resulting in a significant main effect of hemispace [*F*(1,15) = 20.932, *p* < 0.001, ES = 0.583; Figure 2A]. A significant main effect of distance [*F*(1,15) = 21.867, *p* < 0.001, ES = 0.593; Figure 2A] revealed that when constructing the first model participants grasped more blocks from near reachable space than from far reachable space (NEAR = 6.5 ± 1.7 blocks; FAR = 3.5 ± 1.7 blocks). The interaction between hemispace and distance was not significant (*p* > 0.05).

##### Fourth model

When constructing the fourth model, participants grasped more blocks from left space when compared with right space as indicated by a significant main effect of hemispace [*F*(1,15) = 17.790, *p* = 0.001, ES = 0.543; LEFT = 6.3 ± 1.9 blocks, RIGHT = 3.7 ± 1.9 blocks; Figure 2A]. In addition, a significant main effect of distance [*F*(1,15) = 12.023, *p* = 0.003, ES = 0.445; Figure 2A] revealed that participants grasped more blocks from far reachable space when compared with near reachable space (NEAR = 4.0 ± 1.6 blocks, FAR = 6.0 ± 1.6 blocks). Moreover, a significant hemispace by distance interaction [*F*(1,15) = 4.747, *p* = 0.046, ES = 0.240; Figure 2A] indicated that participants differentially grasped blocks from left or right hemispace depending upon whether they were grasping in near or far reachable space. Participants displayed a tendency to grasp more blocks from left space than right space when grasping in near reachable space [LN–RN, *t*(15) = 2.663, *p* = 0.018]. This pattern of preference for blocks from left hemispace was further exacerbated when participants were reaching in far reachable space [LF–RF, *t*(15) = 3.626, *p* = 0.002].

##### Overall

When investigating the overall patterns of space use for grasping during model set construction a significant main effect of hemispace [*F*(1,15) = 28.011, *p* < 0.001, ES = 0.651; Figure 2B] was observed. On average, participants grasped blocks from left space later in model set construction when compared with right space (LEFT = 462.3 ± 46.9, RIGHT = 357.7 ± 46.9). Participants also grasped blocks from far reachable space on average later in model set construction when compared to near reachable space as confirmed by a significant main effect of distance [*F*(1,15) = 14.973, *p* = 0.002, ES = 0.500; NEAR = 372.9 ± 49.5, FAR = 447.1 ± 49.5; Figure 2B]. Although there was not a significant interaction between factors (*p* > 0.05), comparisons between the overall grasp score for the LF quadrant and the overall grasp scores for each of the other three quadrants did reveal that participants grasped blocks from LF space significantly later in model set construction than from LN [*t*(15) = 4.015, *p* = 0.001], RN [*t*(15) = 6.352, *p* < 0.001], or RF [*t*(15) = 4.663, *p* < 0.001] space (Figure 2B).

### Discussion

The purpose of *Experiment 1* was to describe space use during an ecologically valid bimanual visually guided grasping task amongst right-handed participants. The results demonstrated that space use for grasping varied according to hemispace and spatial proximity to the participant. More specifically, when participants had the opportunity to grasp building blocks from any quadrant of space (i.e., Model 1) they preferentially selected blocks from right space; moreover, the majority of blocks were selected from near reachable space. In contrast, participants largely ignored (or, “neglected”) the blocks in LF space until later in model set construction.

It is possible that this pattern of space use may have been influenced by hand dominance and associated biomechanical constraints. One could argue that participants chose to grasp in right hemispace first because that space is closer to their dominant right hand. To examine this possibility, the protocol used in *Experiment 1* was repeated in a group of left-handed participants for *Experiment 2*. If the pattern of grasping observed in *Experiment 1* was a consequence of handedness, it was expected that left-handed participants would display the reverse behavior; that is, participants would choose to grasp from left hemispace first, and would “neglect” right rather than left far space.

## Experiment 2: Left-Handers

In *Experiment 2* the conditions of *Experiment 1* were repeated with left-handed participants.

### Materials and methods

#### Participants

Sixteen self-declared left-handed participants from the University of Lethbridge took part in *Experiment 2* (nine males; 18–35 years). The study was performed with approval by the University of Lethbridge Human Subject Research Committee. Written informed consent was provided prior to the initiation of the study. Participants were naïve to the purposes of the study.

#### Apparatus and stimuli

Apparatus and stimuli were identical to those used in *Experiment 1*.

#### Procedures

Procedures were the same as in *Experiment 1*.

#### Data processing and analysis

The data processing and analysis techniques used in *Experiment 1* were repeated for *Experiment 2*.

### Results

#### Handedness questionnaire

Participants had an average handedness questionnaire score of −14.8 ± 11.3. The range of scores was from +9 to −29, two participants reported using their right hand on average more than their left in the selection of activities targeted by the questionnaire. All participants, however, self-identified as being left-handed and all participants used their left hand to fill the questionnaire and sign the consent form.

#### Trial times

A significant main effect of model [*F*(3,45) = 4.203, *p* = 0.011, ES = 0.219] indicated that trial times were significantly affected by the model being constructed. More specifically participants completed the final model (Model 4) significantly faster than the first [*t*(15) = 3.142, *p* = 0.007] model.

#### Hand use for grasping

Left-handed participants used their non-dominant right hand for 45.6 ± 10.0% of all grasps. Interestingly, there was not a significant difference between hands when analyzing contralateral grasps (*p* > 0.05) with participants using their right hand for 4.0 ± 3.7% of grasps to left hemispace and their left hand for 8.4 ± 8.6% of grasps to right hemispace.

#### Space use

##### First model

**Figure 3:**
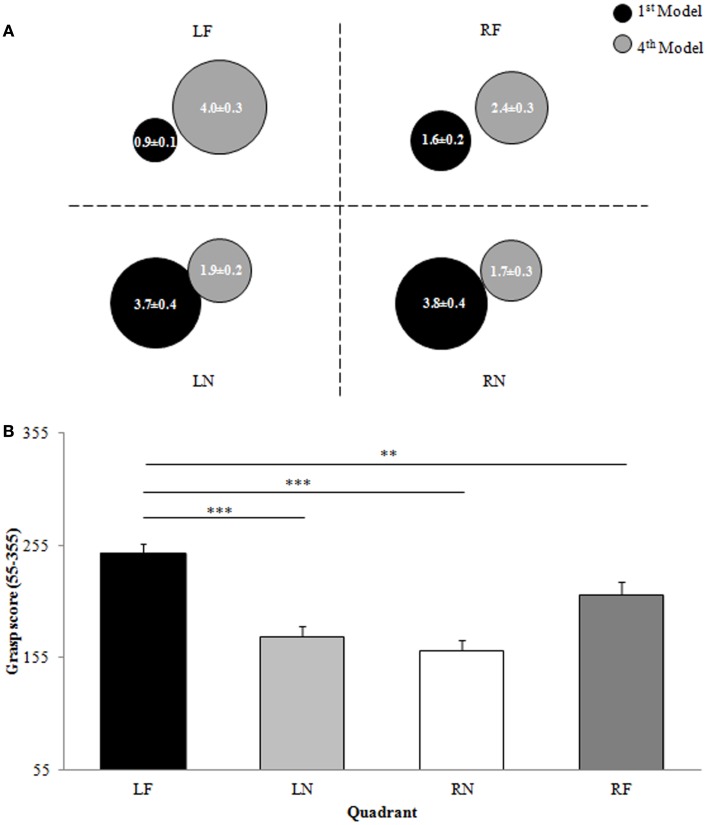
**Experiment 2: left-handers**. **(A)** Representation of the proportion of blocks (0–10) used from each quadrant for the construction of the first and fourth models in the model set. Data are means and standard errors. **(B)** Overall grasp score for each quadrant of reachable space. Data are means and standard errors. LF, left far; LN, left near; RN, right near; RF, right far. *p < 0.05, **p < 0.01, ***p < 0.001 with respect to LF.

A significant main effect of distance [*F*(1,15) = 111.667, *p* < 0.001, ES = 0.917; Figure 3A] revealed that participants grasped significantly more blocks from near compared to far reachable space (NEAR = 7.5 ± 1.3 blocks; FAR = 2.5 ± 1.3 blocks) when constructing the first model. There was not a significant main effect of hemispace or a significant hemispace by distance interaction (*p* > 0.05).

##### Fourth model

Participants grasped more blocks from left space compared to right space (LEFT = 5.9 ± 1.9 blocks, RIGHT = 4.1 ± 1.9 blocks) when constructing the fourth model as indicated by a significant main effect of hemispace [*F*(1,15) = 10.970, *p* = 0.005, ES = 0.422; Figure 3A]. In addition, left-handed participants grasped significantly more blocks from far reachable space than near reachable space [*F*(1,15) = 14.195, *p* = 0.002, ES = 0.486; NEAR = 3.6 ± 1.9, FAR = 6.4 ± 1.9; Figure 3A]. Moreover, block selection from near and far reachable space was differentially affected by whether the block was being grasped in left or right space, as indicated by a significant hemispace by distance interaction [*F*(1,15) = 5.400, *p* = 0.035, ES = 0.265; Figure 3A]. When reaching to near space participants did not differentially grasp blocks from left or right hemispace (LN–RN, *p* > 0.025); however, when reaching to far space participants grasped more blocks from left space when compared to right space [LF–RF, *t*(15) = 3.014, *p* = 0.009].

##### Overall

A significant main effect of hemispace [*F*(1,15) = 5.807, *p* = 0.029, ES = 0.279; Figure 3B] revealed that participants grasped blocks from left hemispace on average later in model set construction than blocks located in right hemispace (LEFT = 436.2 ± 54.6, RIGHT = 383.8 ± 54.6). In addition, a significant main effect of distance [*F*(1,15) = 60.472, *p* < 0.001, ES = 0.801; Figure 3B] indicated that participants grasped blocks from far reachable space on average later in model set construction than those located in near reachable space (NEAR = 347.1 ± 45.7, FAR = 372.9 ± 45.7). Moreover, a hemispace by distance interaction that approached significance [*F*(1,15) = 4.123, *p* = 0.060, ES = 0.216; Figure 3B] suggested that participants differentially grasped blocks from near and far reachable space depending on whether the blocks were being grasped from left or right hemispace. More specifically, the overall grasp score was significantly higher in LF space than LN [*t*(15) = 7.588, *p* < 0.001], RN [*t*(15) = 7.015, *p* < 0.001], and RF [*t*(15) = 3.093, *p* = 0.007] space, indicating that on average participants grasped blocks from left far space later in model set construction than the blocks elsewhere in space (Figure 3B) just as right-handers did.

#### Comparison between right- and left-handed participants

A three-factor RM ANOVA was conducted to assess the effect of hand dominance on space use as determined by the overall grasp score. Hemispace (LEFT, RIGHT) and distance (NEAR, FAR) were within-subjects factors, *Experiments 1* and *2* were the between subject factor. A significant distance by experiment interaction [*F*(1,30) = 4.246, *p* = 0.048, ES = 0.124] indicated that the experiment (and consequently the participants handedness) influenced space use with respect to whether the participant was grasping in NEAR or FAR reachable space. Specifically, participants in *Experiment 2* (left-handed) grasped blocks from NEAR reachable space on average earlier in model set construction than participants in *Experiment 1* (right-handed). The interactions between hemispace and experiment, and distance, hemispace, and experiment were not significant (*p* > 0.05) implying that the “neglect” of LF space was not a product of hand dominance.

#### Correlation analysis for Experiments 1 and 2

To further investigate whether the tendency to neglect the LF space was a product of using the left hand less often for grasping, a bivariate correlation analysis was conducted between the overall grasp score for space use in LF space and the average left hand use for grasping on the data from all participants (right- and left-handed). Right-handed participants grasped with their left hands 29.8 ± 13.8% of the time, in contrast with left-handers who used their left hands for 54.2 ± 9.9% of grasps. The overall correlation between left hand use and LF space was not significant (*p* > 0.05), suggesting that the neglect of LF space is not related to hand use. In addition, a correlation analysis between the overall grasp score for LF space and the handedness questionnaire score was not significant (*p* > 0.05).

### Discussion

The second experiment was designed to investigate whether “neglect” of LF reachable space was a consequence of handedness. In other words, because the participants in *Experiment 1* were all right-handed, one could argue that this hand preference was the cause of the observed LF neglect. Surprisingly, left-handed participants behaved much as right-handers. Both right- and left-handed participants delayed grasping in LF space generally toward the end of the task. This finding (and the fact that there was no correlation between overall LF space use and left hand use for grasping or handedness questionnaire score) suggests that the observed spatial biases were not simply a product of biomechanical constraints resulting from hand dominance.

The phenomenon that young healthy adults display an inherent tendency to neglect LF reachable space during grasping expands our current knowledge of visuospatial processing in general, but also has implications for our understanding of visuomotor deficits in a variety of patient populations. In the first instance it is necessary to elucidate the basis of these spatial biases. One possibility worthy of investigation was that the pattern of space use was a consequence of attentional biases. Accordingly, the experimental protocol was repeated with participants wearing eye tracking glasses (coupled with a scene camera) to provide an inference of the direction of visual attention during the reaching-to-grasp task. We hypothesized that if the neglect of LF space during grasping was a consequence of inattention, then the patterns of gaze would closely mirror those of grasping (i.e., participants would not direct visual attention to left far space until later in model set construction).

## Experiment 3: Eye Tracking

In *Experiment 3* the protocol used in *Experiments 1* and *2* was repeated in a population of right-handed participants fitted with eye tracking glasses to provide information on gaze position.

### Materials and methods

#### Participants

Twelve self-reported right-handed participants were recruited for this study (three males; 18–35 years). The University of Lethbridge Human Subject Committee approved the study. All participants provided written informed consent prior to participation in the study. Participants were naïve to the nature of the study.

#### Apparatus and stimuli

##### Handedness questionnaire

The handedness questionnaire used was the same as that used in *Experiments 1* and *2*.

##### Building block task

The building block task that was used was the same as that used in *Experiments 1* and *2* with the exception that participants constructed only one set of four models (using MEGA BLOKS^®^) in the task.

##### Eye tracker

Participants were fitted with head-mounted eye tracking glasses (Eyelink II^®^; SR Research, Osgoode, ON, Canada) with a scene camera mounted anteriorly near the center of the headband. The eye tracking glasses allow 3D eye tracking whilst the addition of the scene camera enables the overlay of gaze position onto the outward scene video (collected at 30 Hz).

#### Procedures

The procedures were the same as those used in *Experiments 1* and *2* with the exceptions that the workspace consisted of a tabletop (70 L × 120 W × 74 cm H) surrounded on three sides by black partitions and walls. The eye tracker was fitted to the participant and calibrated according to manufacturer recommendations. Following calibration participants were requested to close their eyes while the building blocks were distributed appropriately on the tabletop. The first model to be replicated was placed on a base plate located centrally at the far border of the workspace between left and right space (Figure 1A). Once data recording was initiated, participants were instructed to open their eyes and use the available blocks to replicate the displayed model as quickly and accurately as possible on a second base plate (19 L × 19 W cm) located centrally immediately in front of them (at the intersection of right and left space; Figure 1A). Following completion of the model, participants were asked to close their eyes while both models were removed and a new model to be replicated was provided. Each participant constructed the same four models; model order was randomized between participants.

Gaze position and model construction were recorded for subsequent analysis using the eye tracker and associated scene camera.

#### Data processing and analysis

All recordings were analyzed offline. The videos were cropped into individual trials (i.e., Models 1–4) using the events of the eyes opening and the final release of the constructed model with both hands. The scene videos for the first and fourth model were manually scored to provide the number of blocks (0–10 blocks) grasped from each quadrant for these models. In addition, a more detailed indication of space use was provided by numbering each grasp (1–40) as described in *Experiment 1*.

Each frame of gaze position data was also manually scored as being allocated to a particular quadrant of space, the “home” model and plate, or the “build” model and plate to provide an inference of overt visual attention. The initial gaze position (not directed toward the home or build models) during construction of Model 1 was recorded for each participant. The relative proportion of the trial during which gaze was directed to each of the quadrants was calculated [(frames with gaze located in a specific quadrant/overall frames that gaze position was located in any of the four quadrants) × 100] for each of the four models. Gaze directed to the home and build models and plates was excluded from the analysis.

Data analysis procedures were the same as those used in *Experiments 1* and *2* with the exceptions that the initial gaze position data (for Model 1) was summarized descriptively and gaze position data for the first and fourth models and over the course of the complete model set were entered into separate two-factor (hemispace × distance) RM ANOVAs, with hemispace (LEFT, RIGHT), and distance (NEAR, FAR) as within-subject factors.

### Results

#### Handedness questionnaire

Participant handedness was determined by the modified handedness questionnaire, with the average score of +32.3 ± 5.2 (scores ranging from +20 to +38) confirming that all participants were right-handed.

#### Trial times

A main effect of model approaching significance [*F*(3,33) = 2.783, *p* = 0.056, ES = 0.202] suggested that trial time was affected by the model being constructed, follow-up comparisons however, failed to reach significance (*p* > 0.017).

#### Hand use for grasping

Participants used their right hand for 60.0 ± 11.9% of all grasps. The analysis of contralateral grasps revealed that participants made significantly more contralateral grasps with the right hand to left hemispace (11.9 ± 10.1%) when compared to grasps made with the left hand to right hemispace [1.9 ± 2.6%; *t*(11) = 2.907, *p* = 0.014].

#### Space use

##### First model

**Figure 4:**
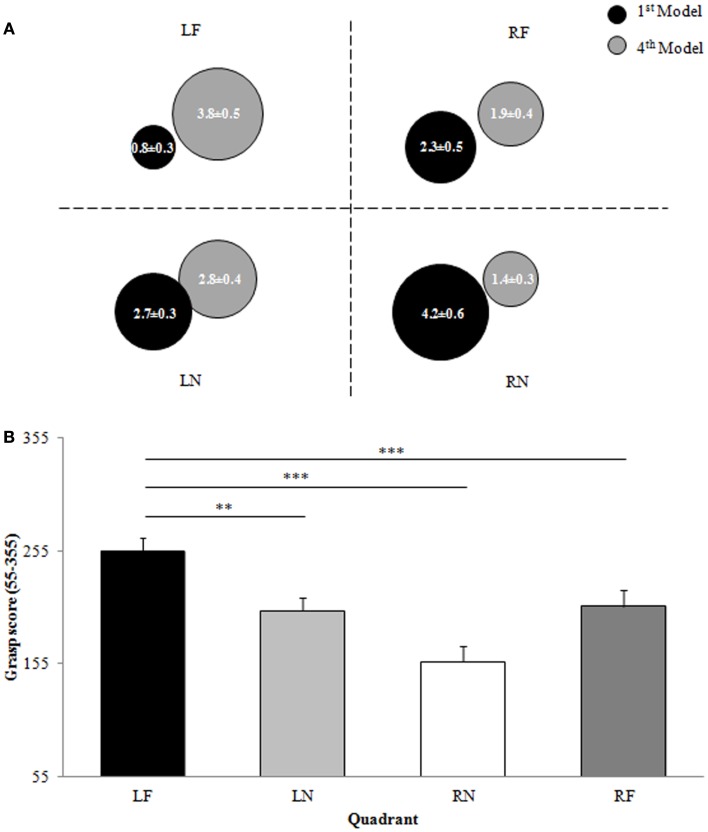
**Experiment 3: eye tracking**. Space use: **(A)** Representation of the proportion of blocks (0–10) used from each quadrant for the construction of the first and fourth models in the model set. Data are means and standard errors. **(B)** Overall grasp score for each quadrant of space. Data are means and standard errors. LF, left far; LN, left near; RN, right near; RF, right far. *p < 0.05, **p < 0.01, ***p < 0.001 with respect to LF.

When constructing the first model in the set, participants grasped more blocks from right space than left space (LEFT = 3.5 ± 1.2 blocks, RIGHT = 6.5 ± 1.2 blocks) as confirmed by a significant main effect of hemispace [*F*(1,11) = 17.471, *p* = 0.002, ES = 0.614; Figure 4A]. In addition, a main effect of distance [*F*(1,11) = 7.694, *p* = 0.018, ES = 0.412; Figure 4A] indicated that participants grasped more blocks from near space when compared to far space (NEAR = 6.8 ± 2.3 blocks, FAR = 3.2 ± 2.3 blocks). The hemispace by distance interaction was not significant (*p* > 0.05).

##### Fourth model

A significant main effect of hemispace [*F*(1,11) = 11.957, *p* = 0.005, ES = 0.521; Figure 4A] revealed that participants grasped significantly more blocks from left hemispace than from right hemispace (LEFT = 6.7 ± 1.7 blocks, RIGHT = 3.3 ± 1.7 blocks) to construct the fourth model in the model set. Space use for grasping during the construction of the fourth model was not, however, significantly influenced by distance (*p* > 0.05). Participants grasped 4.3 ± 2.1 blocks from near space and the remaining 5.7 ± 2.1 blocks from far space. In addition, the hemispace by distance interaction was not significant (*p* > 0.05).

##### Overall

When analyzing space use for grasping across the construction of all four models it was found that participants grasped blocks from left hemispace on average later in model set construction than blocks in right hemispace, as indicated by a significant main effect of hemispace [*F*(1,11) = 13.128, *p* = 0.004, ES = 0.544; LEFT = 457.2 ± 44.8, RIGHT = 362.8 ± 44.8; Figure 4B]. A significant main effect of distance [*F*(1,11) = 6.374, *p* = 0.028, ES = 0.367; Figure 4B] revealed that participants grasped blocks in far space on average later than those in near space (NEAR = 359 ± 70.6, FAR = 461 ± 70.6). The hemispace by distance interaction failed to reach significance (*p* > 0.05), however, planned comparisons between the overall grasp score for LF space and the overall grasp score for each of the other three quadrants of space (as per *Experiments 1* and *2*) revealed that participants grasped blocks from LF space on average later in model set construction than from LN space [*t*(11) = 2.882, *p* = 0.015], RN space [*t*(11) = 4.063, *p* = 0.002], or RF space [*t*(11) = 3.169, *p* = 0.009; Figure 4B].

#### Gaze position

**Figure 5:**
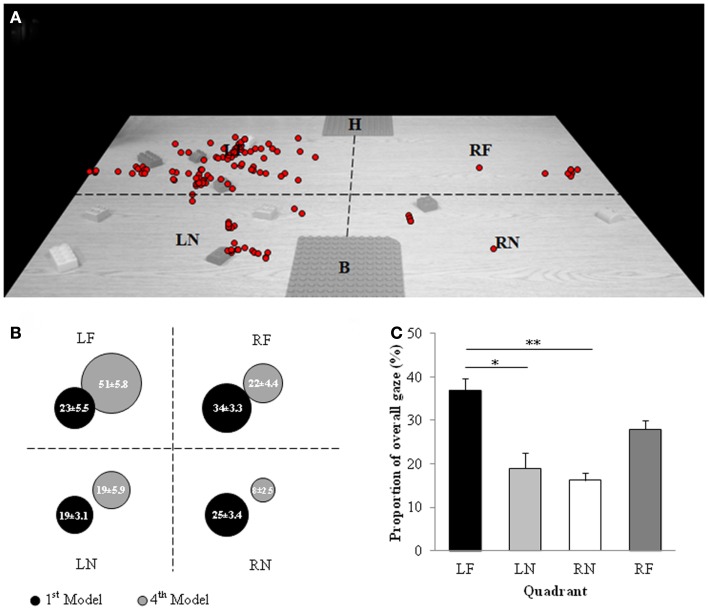
**Experiment 3: eye tracking**. Gaze position: **(A)** Example of gaze position data during construction of the fourth model for a single participant. Each red circle represents gaze position for a single frame of video. Dashed black lines notionally divide workspace into quadrants; left near (LN), left far (LF), right near (RN), and right far (RF). The model to be replicated is located at the far “home” base plate “H” and model being constructed is located at the near “build” base plate “B.” **(B)** Representation of the proportion of gaze (%) directed toward each quadrant of reachable space during construction of the first and fourth models in the model set. Data are means and standard errors. **(C)** Overall proportion of gaze directed to each quadrant of space. Data are means and standard errors. *p < 0.05, **p < 0.01, ***p < 0.001 with respect to LF.

Gaze position data for one participant was discarded due to equipment failure. An example of gaze position data during construction of the fourth model for one participant is provided in Figure 5A.

##### First model

Gaze position for the remaining 11 participants was not significantly affected by hemispace or distance (*p* > 0.05) during the construction of the first model. Furthermore, the hemispace by distance interaction was not significant (*p* > 0.05).

##### Fourth model

A significant main effect of hemispace [*F*(1,10) = 33.588, *p* < 0.001, ES = 0.771; Figure 5B] was observed during construction of the fourth model in the set, with participants spending a higher proportion of model construction time with gaze positioned in left hemispace as compared to right hemispace (LEFT = 69.9 ± 11.4%, RIGHT = 30.1 ± 11.4%). In addition, when constructing the fourth model participants spent more time with gaze positioned in far space as compared to near space (NEAR = 27.0 ± 20.3%, FAR = 73.0 ± 20.3%) as indicated by a significant main effect of distance [*F*(1,10) = 14.149, *p* = 0.004, ES = 0.586; Figure 5B]. The hemispace by distance interaction was not significant (*p* > 0.05).

##### Overall

A significant main effect of hemispace [*F*(1,10) = 4.741, *p* = 0.054, ES = 0.322; Figure 5C] revealed that on average participants directed gaze more toward left hemispace than right hemispace (LEFT = 55.8 ± 8.9%, RIGHT = 44.2 ± 8.9%) across the construction of all four models. Furthermore, a significant main effect of distance [*F*(1,10) = 17.180, *p* = 0.002, ES = 0.632; Figure 5C] was reported with gaze position being directed toward near space for 35.2 ± 11.9% of model set construction and far space for the remaining 64.8 ± 11.9%. The hemispace by distance interaction was not significant (*p* > 0.05). The fact that gaze was predominantly directed toward left space across the construction of the model set suggests that during construction of the second and/or third models participants must have allocated overt attention predominantly to left hemispace. Analysis using a 2 (hemispace) × 2 (distance) × 4 (Model) RM ANOVA revealed a significant hemispace by model interaction [*F*(3,30) = 11.338, *p* < 0.001, ES = 0.531]. More specifically, during construction of the first two models participants allocated gaze equally to left and right hemispace (*p* > 0.05), however, for construction of the third and fourth models, participants spent significantly more time with gaze allocated to left hemispace [Model 3, *t*(10) = 3.721, *p* = 0.004; Model 4, *t*(10) = 5.795, *p* < 0.001]. Finally, to investigate whether the tendency to direct gaze to left hemispace resulted from participants directing gaze to LF space planned pairwise comparisons between LF space and LN, RN, and RF space were conducted. Results indicated that participants directed their gaze to the LF quadrant for a significantly larger proportion of model construction than LN space [*t*(10) = 3.096, *p* = 0.011] and RN space [*t*(10) = 4.210, *p* = 0.002]. Participants displayed a tendency to direct gaze toward LF space more than RF space, however, this difference failed to reach significance [*t*(10) = 1.924, *p* = 0.083; Figure 5C].

#### Initial gaze position

Initial gaze position was located in LN space for 18.2% of participants, LF space for 27.3% of participants, RN space for 9.1% of participants, and the RF quadrant for the remaining 45.5% of participants. To gain an inference of whether gaze was directed to the quadrant where the first block would be grasped from or rather whether the participants surveyed their options prior to initiating grasping, a Cramer’s V test between initial gaze position and initial grasp location was completed. The Cramer’s V analysis was not significant (*p* > 0.05), suggesting participants did not initially locate their gaze to the quadrant from which they would grasp the first block.

### Discussion

Overall patterns of space use were very similar between *Experiments 1* and *3*, with right-handed participants grasping blocks from LF space on average later in model set construction than blocks in the other three quadrants. As anticipated, the overall pattern of gaze position paralleled the pattern of grasping, particularly during construction of the fourth model with gaze being directed to task relevant locations. More specifically, visual attention appeared to be fairly evenly distributed between quadrants early in model set construction when participants were presumably surveying the workspace assessing their options, however, by the fourth model participants’ dedicated considerable visual attention to LF space. Whilst this finding might not be surprising, as the majority of the remaining blocks were located in the LF quadrant what was perhaps surprising were the results from the initial gaze analysis. Previous studies have shown that grasping is preceded by eye movements toward the object to be grasped (46–51). The results of the initial gaze analysis however, showed no relationship between initial gaze position and initial grasp location. This finding suggests a dissociation between gaze and grasp and will be discussed in more detail in the general discussion. The findings from *Experiment 3* do provide support for the notion that the spatial biases, specifically the neglect of LF space, observed amongst right- and left-handed participants during the reaching-to-grasp task may be attentional in origin; this possibility will be further discussed in the following section.

## General Discussion

We assessed space use for grasping during a bimanual visually guided reach-to-grasp task amongst healthy adults. In addition, we characterized the patterns of gaze position throughout the grasping task to provide an inference of overt visual attention. Participants were required to locate, grasp, and orient to specific building blocks available on a tabletop in order to replicate a series of complex 3D models. The tabletop was notionally divided into equal sized quadrants differentiated by left and right hemispace, as well as near and far reachable space. The building blocks necessary to construct each model were available in each of the quadrants. Participants displayed a tendency to “neglect” left far space until the final stages of the task. Moreover, similar patterns of space use were observed for both right- and left-handed participants suggesting that the patterns of space use for grasping were not simply a result of hand dominance and associated biomechanical constraints. Despite a dissociation between initial gaze position and initial grasp location, the overall gaze position data largely corresponded with patterns of grasp (i.e., participants overt attention was directed toward the space from which participants were grasping building blocks) which highlights the possibility that the observed “neglect” of left far space may partially be a result of inherent attentional biases.

To our knowledge, this is the first study to characterize space use during a natural visually guided bimanual grasping task in healthy adults. The findings of this study expand our understanding of spatial cognition in humans. This knowledge has thus far been largely garnered from studies on non-human species or alternatively from studies that have utilized standardized paper-and-pencil or computerized assessments to test specific aspects of spatial attention and perception, spatial memory and/or mental imagery (52, 53). Though these common assessments have provided a wealth of knowledge on spatial biases in healthy, aging, and patient populations, the tasks are typically presented in two-dimensional space (i.e., computer monitor) and/or are unimanual and are therefore not truly representative of the bimanual object interactions that we complete hundreds of times each day. As such, these standardized tasks do not address the question of whether equivalent spatial biases are present in real-world grasping tasks. By developing an ecologically valid bimanual task that has well characterized motor and perceptual demands (i.e., replicable visuomotor requirements, visuospatial complexity) it has been possible to add to our understanding of the spatial biases that occur in complex, multi-factorial tasks typically encountered in the everyday environment.

We found that when constructing the first model in the model set (i.e., when there was equal opportunity to grasp blocks from all quadrants) right-handed participants preferentially grasped blocks from right hemispace. Left-handed participants also selected marginally more pieces from right hemispace when compared to left hemispace (5.4 blocks from right hemispace, 4.6 blocks from left hemispace); however, it should be noted that this differential pattern of lateral space use was not significant. All participants grasped the majority of blocks from near reachable space (i.e., reachable without movement of the trunk). Whilst the spatial biases observed relating to distance likely reflect the biomechanical efficiency and comfort of grasping targets in closer proximity to the body (i.e., shorter movement trajectory) we suggest that the lateral spatial biases may be influenced by hand preference. It has previously been reported that right-handed individuals use their dominant hand almost exclusively when grasping objects in ipsilateral space (i.e., right hemispace) or at the body midline (54–56). Furthermore, a strong right hand preference remains when right hand dominant individuals reach to contralateral space. In contrast, left-handed individuals have a tendency to use their dominant and non-dominant hands more equally, normally using the hand ipsilateral to the object for grasping (41, 57–59). In agreement with these earlier studies, the participants in *Experiment 1* grasped approximately 71 ± 14% of the blocks for the first model with their dominant right hands. In contrast, the left-handed participants in *Experiment 2* grasped around 46 ± 10% of the blocks with their non-dominant hands, moreover, 91% of these grasps with the right hand were in right hemispace. Although it remains possible that the “neglect” in LF space is due exclusively to biomechanical constraints, it is unlikely. First, assuming that left-handers by definition are more skilled with their left hand, one would have expected this group to show the opposite pattern of space use to right-handers and therefore neglect RF space. This was not the case, however, with left-handers showing a similar pattern of neglect of LF space to right-handers. Second, there was no correlation between left hand use and the overall grasp score (space use) in the LF quadrant. This finding strongly suggests that hand preference for grasping did not influence participants’ space use with respect to the LF quadrant. Finally, investigations of kinematics of left- and right-handed reach-to-grasp movements have revealed, at most, minimal differences between hands (60–63) suggestive that the preference to use the right hand (particularly in right space) is not driven by a kinematic advantage.

In stark contrast to the pattern of space use observed during construction of the first model, when constructing the fourth and final model in the series participants grasped the majority of blocks from the far left quadrant where the majority of the remaining blocks were located. The finding that LF space was largely “neglected” until alternative spatial locations had been exhausted was confirmed by the overall grasp score data. This “neglect” of LF space until later in model set construction would appear to be somewhat intuitive for right-handed participants based upon the biomechanical inefficiency associated with the longer movement trajectory to make grasps to far contralateral space with the dominant hand, or alternatively the necessity of using the non-dominant ipsilateral hand. Indeed, this would be consistent with the literature (64–66) suggesting that contralateral movements are computationally more complex and therefore presumably more effortful for the participant. Again, however, the pattern of space use for grasping was largely consistent between right- and left-handed participants. As left-handers typically reach to left far space with their dominant hand it appears that biomechanical inefficiencies cannot fully explain the “neglect” observed. In contrast, the spatial biases for grasping seen in the current studies are consistent with numerous studies that have found that neurologically healthy adults tend to display a rightward bias in bisection performance when viewing lines in extrapersonal space (21–25). Gamberini et al. (22) for example, presented participants with lines at four viewing distances (two in peripersonal space, two in extrapersonal space) in both real and virtual environments. Participants displayed an abrupt left-to-right shift of bisection upon transitioning from peripersonal to extrapersonal space in both environments. Despite the entirety of the current task being completed in reachable space, it should be noted that in our experiments a leftward bias, characteristic of the *pseudoneglect* exhibited by healthy adults (18, 19) was not observed when participants were grasping in near reachable space.

The gaze position data collected during the same reaching-to-grasp task provides additional insight into the spatial biases observed amongst the right- and left-handed participants and suggests the possibility that the patterns of space use for grasping may have their origins in visual attentional biases. During construction of the first model participants’ gaze appeared to be fairly evenly distributed in all quadrants. We speculate that the lack of spatial bias during the construction of the first model results from the novelty of the workspace and task. This postulation was further supported by inspection of the initial gaze position data, which indicated a dissociation between gaze position and initial grasp location. Initially, gaze was predominantly directed to RF space whilst the participants’ initial grasp tended to be located in LN space. In agreement with the literature (67–70) the participants appeared to scan the workspace to locate the salient blocks prior to initiating construction rather than use memory of spatial location. Furthermore, participants did not limit their search to the favored area of grasping (i.e., RN space). During the construction of subsequent models, however, as may be expected the spatial distribution of the gaze position data largely mirrored that observed in the grasping behavior with gaze being directed toward the task relevant locations (46–51). Specifically, when constructing the fourth model, participants directed overt visual attention predominantly toward left hemispace and far space, this corresponds with the location of the majority of the blocks remaining in the array (as well as the spatial biases observed during grasping). Interestingly, the overall gaze position data (i.e., across all four models) indicated that on average participants dedicated a greater proportion of overt visual attention to left hemispace and far space.

A possible explanation for the increased visual attention to left hemispace in the right-handed participants is that when grasping blocks from left space participants would either be using their dominant right hand to reach and grasp in contralateral space, or alternatively would be using their non-dominant hand to grasp the block in ipsilateral space. It is conceivable that both of these scenarios would be more attentionally demanding for the participant than using the dominant hand to reach in ipsilateral space. Therefore, we may expect that participants would allocate more attentional resources (i.e., gaze) to effectively and efficiently grasp blocks in left hemispace. Further inspection of the gaze position data presented the possibility that the participants’ overt visual attention may have been drawn toward far space by the placement of the “home” model. This postulation is in agreement with prior work (67–70) suggesting that one of the two major functions of the eyes during everyday actions is to gather information on objects with which we are interacting (*locating* and *checking*). In the case of the model building task this would necessitate frequently checking the “home” model to identify the next block to be located as well as to ensure the accuracy of the replica model. Despite the exclusion of gaze directed to the “home” model or base plate from our analyses, the “home” model may have attracted the participants gaze to far space. We intend to examine these possibilities in future studies to elucidate the basis of the observed spatial biases described here. Despite the reported natural propensity for gaze to be drawn toward left far space the finding that the general pattern of gaze followed that of grasping provides support for the postulation that the observed patterns of space use for grasping result from attentional biases.

Our findings have implications for our understanding of visuomotor deficits in a variety of patient populations, particularly those with hemispatial neglect. The data suggests that neurologically intact individuals physically neglect left far space, potentially as a consequence of inattention to this spatial location. This raises the possibility that the spatial biases observed among individuals with left hemispace neglect (i.e., bias toward right hemispace) may not purely be a result of syndrome specific neglect but may reflect in part an exacerbation of an inherent tendency to neglect left far peripersonal space. Alternatively, it is possible that the findings could be explained by hemispheric specialization for visually guided grasping. Neuroimaging studies have revealed several brain areas implicated in the planning and execution of human [i.e., superior parieto-occipital cortex (SPOC); (71, 72)] and primate [i.e., V6A; (73, 74)] visually guided grasping. Furthermore these studies have highlighted the unique role of the posterior parietal cortex in coding reachable vs. unreachable space (71, 72, 75). For example, Gallivan et al. (72) found that SPOC was selectively activated for objects within reachable space. Interestingly, this activation was found in the left hemisphere for both right- and left-handers. If SPOC in the left hemisphere turns out to be specialized for distinguishing object within reach then, one might expect objects within right hemispace to be preferentially discriminated. This bias could account for the late use of LF space for grasping. Future research should aim to elucidate the basis of the neglect of left far space with respect attentional biases and/or hemispheric specialization. In addition, the contribution of this inherent neglect of left far space to the visuomotor deficits observed in patients with right hemisphere lesions with and without unilateral visuospatial neglect warrants further investigation.

## Author Contributions

Conception and design of study: Claudia L. R. Gonzalez and Devon C. Bryant. Provision of study materials and analysis tools: Claudia L. R. Gonzalez. Collection of Data: Devon C. Bryant and Natalie de Bruin. Analysis and interpretation of data: Natalie de Bruin and Claudia L. R. Gonzalez. Drafting and revision of manuscript: Natalie de Bruin and Claudia L. R. Gonzalez.

## Conflict of Interest Statement

The authors declare that the research was conducted in the absence of any commercial or financial relationships that could be construed as a potential conflict of interest.
